# Quality of Life Assessment With European Organisation for Research and Treatment of Cancer Questionnaire (Head and Neck Module 43) and Its Clinicopathological Correlation Among Patients Treated for Oral Squamous Cell Carcinoma: An Exploratory Study

**DOI:** 10.7759/cureus.34650

**Published:** 2023-02-05

**Authors:** Karthikeyan Ramalingam, Murugesan Krishnan, Pratibha Ramani, Arvind Muthukrishnan

**Affiliations:** 1 Oral Pathology and Microbiology, Saveetha Dental College and Hospitals, Saveetha Institute of Medical and Technical Sciences, Chennai, IND; 2 Oral and Maxillofacial Surgery, Saveetha Dental College and Hospitals, Saveetha Institute of Medical and Technical Sciences, Chennai, IND; 3 Oral Medicine and Radiology, Saveetha Dental College and Hospitals, Saveetha Institute of Medical and Technical Sciences, Chennai, IND

**Keywords:** exploratory study, prospective cohort, hn43, eortc, patient reported outcome measures, oral cancer, quality of life, oscc, prognosis, oral squamous cell carcinoma

## Abstract

Introduction

Oral cancer has a great impact on quality of life (QOL). Many risk factors influence the overall QOL. Our study was performed to evaluate the QOL among patients with oral cancer and to correlate it with age, gender, tobacco usage, and clinicopathological details.

Methods

We have used the European Organisation for Research and Treatment of Cancer Quality of Life Questionnaire Head and Neck Module (EORTC QLQ-HN43) and the Quality of Life Questionnaires for Core 30 (QLQ-C30) among the patients diagnosed with oral cancer after reporting to our institution. The Gpower calculation based on differences between two independent means reported by Meera et al. had a total sample size of 28 with an actual power of 0.9616. Thirty-five patients were included in the present study. Ethical clearance for this study was obtained, and there were no gender or age limits for enrollment. The patient demographic details and case history with relevant treatment information were collected from the DIAS (Dental Information Archival Software) of Saveetha Dental College, Chennai. After obtaining informed consent from the patients, the EORTC QLQ-HN43 and QLQ-C30 questionnaires were given to them. It was used both in Tamil and English. Various domains such as pain, appearance, and oral function were documented. The findings were correlated with clinical and histopathological findings. The collected data were tabulated and statistically analyzed with IBM Statistical Package for the Social Sciences (SPSS) version 20 (IBM Corp., USA). The mean ± SD were calculated for continuous variables, and frequency with percentage was determined for categorical parameters.

Results

The study included both men (57%) and women (43%) in the age range of 30-70 years, with a mean age of 50 years. Study samples included tobacco users (82%) and non-tobacco users (18%). Out of the 35 patients, 15 patients had lesions involving the buccal mucosa (42%) and 10 involving the tongue (28%). Oral squamous cell carcinoma (OSCC) was the most common type of lesion, and it was mostly treated surgically with resection and excision (82%), or just excision (18%). Seventy percent of our patients underwent reconstruction, while primary closure was done in only 30% of cases. All of the patients underwent neck dissection, including supraomohyoid neck dissection (52%), modified radial neck dissection (40%), and radial neck dissection (8%). Histopathology revealed that 49% had well-differentiated squamous cell carcinoma, 23% had moderately differentiated squamous cell carcinoma, and 28% had poorly differentiated squamous cell carcinoma. Out of the 35 included cases, five patients had died (14%). The primary site was buccal mucosa in all five cases, and surprisingly, three patients also had recurrences post-surgery or post-radiotherapy. We observed that the average rating of overall health and overall QOL at the time of diagnosis was 5.4. After one year of follow-up, the average rating of overall health and overall QOL was found to be 3.4.

Conclusion

The administration of EORTC QLQ-HN43 was found to be efficacious in our study on patients with OSCC. We could identify baseline data regarding the QOL of our patients treated for OSCC. We have identified critical domains of oral function that need to be focused upon to improve the overall QOL of OSCC patients through adjunctive therapies. We have also identified higher mortality and overall poorer QOL in patients with OSCC involving the buccal mucosa.

## Introduction

In the current global scenario, the treatment that prioritizes the autonomy of the patient is the most desired modality. Even in the treatment of patients with oral cancer, there is a trend toward moving from conventional outcomes of survival and response rate to personalized management that encompasses multiple outcomes [1].

Statistics reveal that cancers of the oral cavity and lips rank sixth among global malignancies, with a reported mortality rate of 10.2%. Most patients report only in advanced stages, as they have difficulties opening their mouths and swallowing. They also show fear and anxiety about future treatment and survival after a cancer diagnosis [2, 3]. Forty percent of oral cancer cases have been reported in the tongue, involving the postero-lateral border and ventral surfaces. Other major sites include the floor of the mouth, gingival-alveolar sulcus, buccal mucosa, and palate [3, 4].

The concept of "quality of life" (QOL) was introduced by Hecksher and adopted as a keyword by the US National Library of Medicine in 1977. The WHO has defined "quality of life" as an individual’s perception of their position in life in the context of the culture in which they live and in relation to their goals, expectations, standards, and concerns. Beyond traditional health indicators like mortality and morbidity, there has been a recent shift in the focus of health measurement. The incorporation of indicators to identify the impact of illness and impairment on everyday activities and behavior, subjective health, and disability or functional status is essential for holistic treatment planning [3, 5].

The WHOQOL-100 has 100 questions in various domains to assess the respondent’s perceived quality of life (physical capacity, psychological level, level of independence, social relationships, environment, and overall QOL and perception) [6].

The European Organisation for Research and Treatment of Cancer Quality of Life Questionnaire Head and Neck Module (EORTC QLQ-HN43) is a revised and updated version of the Head and Neck Cancer Module (QLQ-HN35). It is a supplementary questionnaire module to be employed in conjunction with the General Quality of Life Questionnaire Core 30 (QLQ-C30). The QLQ-HN43 incorporates twelve multi-item scales to assess pain in the mouth, swallowing, problems with teeth, dry mouth, and sticky saliva, problems with senses, speech, body image, social eating, sexuality, problems with the shoulder, skin problems, and fear of progression. In addition, seven single items assess problems opening the mouth, coughing, social contact, swelling in the neck, weight loss, problems with wound healing, and neurological problems [7].

WHOQOL has 100 questions, and EORTC QLQ-C30 has 30 questions that have to be taken along with 43 questions in QLQ-HN43, which has 73 questions in total. Other questionnaires, like the Oral Health Impact Profile-14 (OHIP-14), are also available but not widely used. Hence, we studied the QOL using the validated EORTC QLQ-HN43 and QLQ-C30 questionnaires. We have compared the overall health score and overall QOL score obtained with the clinical and demographic details of our patients with oral cancer [7].

Limited literature is available regarding QOL among the Indian population, though the oral cancer burden is among the highest in the world [8-10]. This study was designed to record baseline QOL values at the time of diagnosis and QOL values after the one-year follow-up among a cohort of patients with oral cancer. We have attempted to correlate the QOL values with its clinical and histopathological parameters.

## Materials and methods

This prospective analytical exploratory study on a patient cohort with histopathologically proven oral cancers was conducted at Saveetha Dental College, Chennai, India, with QOL measured at the time of diagnosis and another QOL that was performed at least 12 months after primary treatment. Ethical clearance for this study was obtained from the Institutional Ethical Clearance Committee, vide IHEC/SDC/PhD/OPATH-1954/19/TH-001.

The sample size was calculated using G-power and the difference values of independent means were reported in the study by Meera M. et al. [10].

T-tests mean: the difference between two independent means (two groups); analysis a priori: to compute the required sample size, input: tail(s) = 1; effect size d = 1.3262337; α err prob = 0.05; power (1-β err prob) = 0.95; allocation ratio N2/N1 = 1; output: non-centrality parameter δ = 3.5088846; critical t = 1.7056179; Df = 26.

Groups one and two had a sample size of 14 participants each. The total sample size was 28; the actual power was 0.9616846.

The calculated sample size was found to be 28 patients, with an actual power of 0.9616. Thirty-five patients were included in the study. The study period included oral cancer patients reporting to our institution from January 2019 to October 2021. There were no gender or age limits for enrollment. Informed consent was obtained from each participant.

After obtaining informed consent from the patients, the EORTC QLQ-C30 and QLQ-HN43 questionnaires were given to them. The questionnaires were in Tamil and English. Various domains such as pain, appearance, activity, recreation, swallowing, chewing, speech, shoulder mobility, taste, saliva, mood, and anxiety were documented with the health scores and QOL scores.

The patient demographic details and case history with relevant treatment information were collected from the DIAS (Dental Information Archival Software) of our institution. The enrolled patients were followed for a minimum of one year. The EORTC questionnaire was administered again, and the scores were recorded.

Collected data was tabulated with Microsoft Excel and analyzed using the IBM Statistical Package for the Social Sciences (SPSS) version 20 (IBM Corp., USA). Mean ± SD were calculated for continuous variables, and frequency with a percentage was determined for categorical parameters. The QOL values recorded at the time of diagnosis and one year after treatment were also recorded and analyzed.

## Results

This prospective, analytical, and exploratory study on the patient cohort included 35 patients with a confirmed histopathological diagnosis of oral squamous cell carcinoma (OSCC). The included patients were of both male and female gender (57% male, 43% female). They were in the age range of 30-70 years, with a mean age of 50 years. The study samples included tobacco users (82%) and non-tobacco users (18%) (Table 1).

**Table 1:** Gender distribution and tobacco usage among patients with oral squamous cell carcinoma

	Males	Females
Gender	57%	43%
	Users	Non-users
Tobacco	82%	18%

Among tobacco users (28/35), smoking tobacco was identified in 13 patients; tobacco chewing was noted in 13 patients; and two patients smoked and chewed tobacco. Thirteen men smoked tobacco, 12 women and one man chewed tobacco, and two men had both habits.

**Figure 1:**
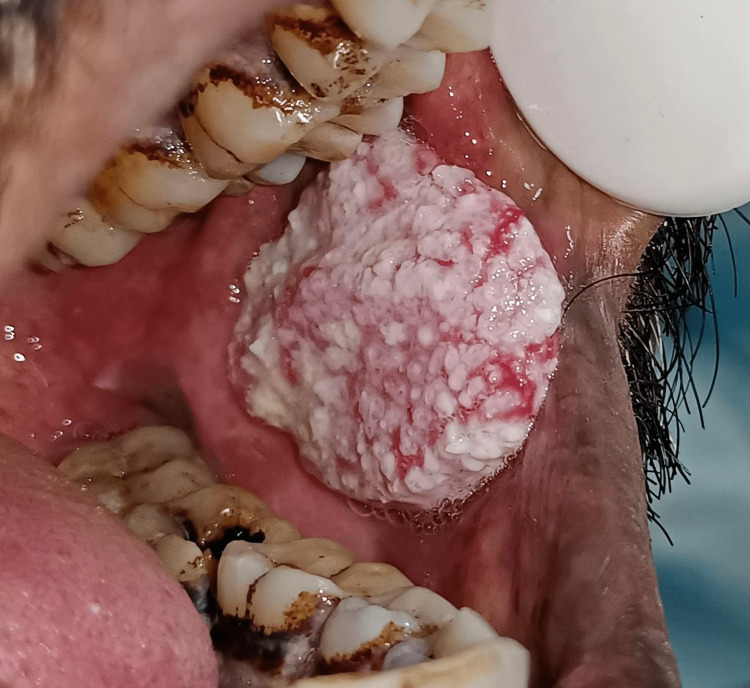
A clinical picture showing a whitish-red proliferative lesion involving the buccal mucosa (well-differentiated squamous cell carcinoma)

**Figure 2:**
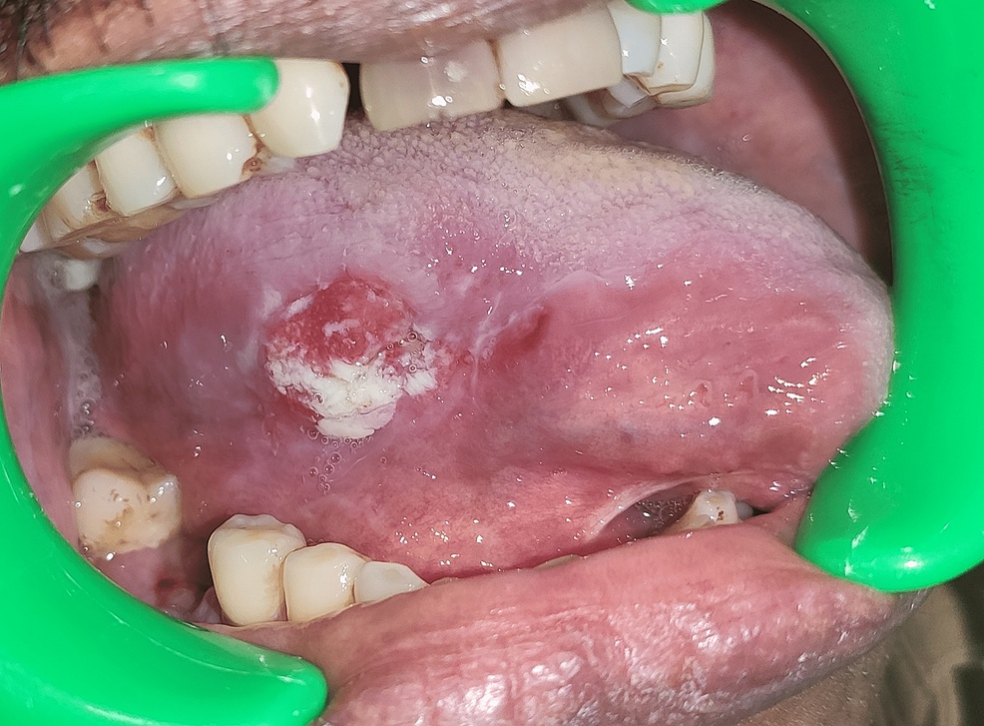
A clinical picture showing a reddish-white proliferative lesion on the lateral border of the tongue (moderately differentiated squamous cell carcinoma)

**Figure 3:**
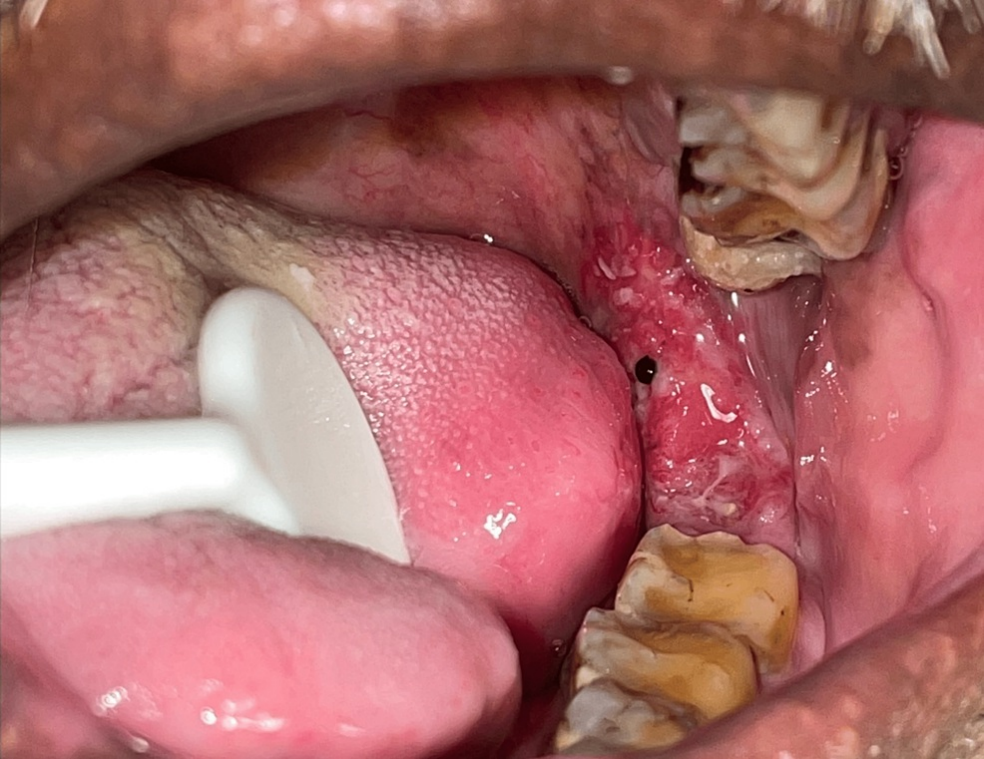
A clinical picture showing an ulcero-proliferative lesion involving the retromolar trigone region (poorly differentiated squamous cell carcinoma)

Out of 35 patients, 33 had T3/T4 tumors with N1/N2 at the time of diagnosis. Fifteen patients had lesions in the buccal mucosa (Figure 1), 10 in the tongue (Figure 2), three in the alveolus (two uppers and one lower), three in the retromolar trigone region (Figure 3), three involving the floor of the mouth, and one in the maxillary tuberosity region extending into the maxillary sinus and oro-pharynx (Table 2).

**Table 2:** The site involvement and number of cases of oral squamous cell carcinoma in our study

Site	Number
Buccal mucosa	15
Tongue	10
Alveolus	3
Retromolar trigone	3
Floor of the mouth	3
Maxillary tuberosity extending into the maxillary sinus and oropharynx	1

Seventy percent of the patients had reconstruction, while the remaining 30% underwent primary closure. Reconstruction included pedicle flaps, myocutaneous flaps from the pectoralis major, and other flaps.

**Figure 4:**
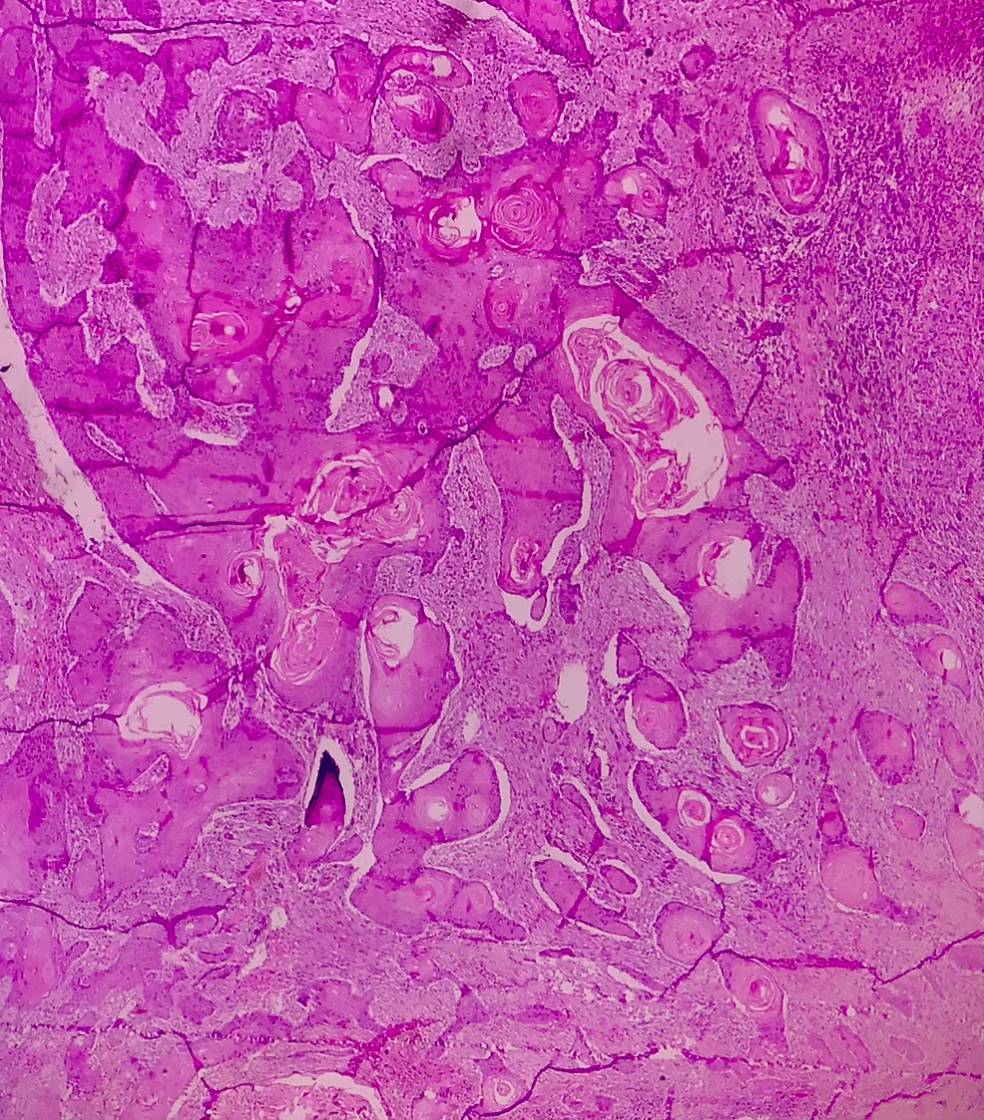
A photomicrograph showing malignant epithelial islands with keratin pearl formation in well-differentiated squamous cell carcinoma (hematoxylin and eosin, 10x)

**Figure 5:**
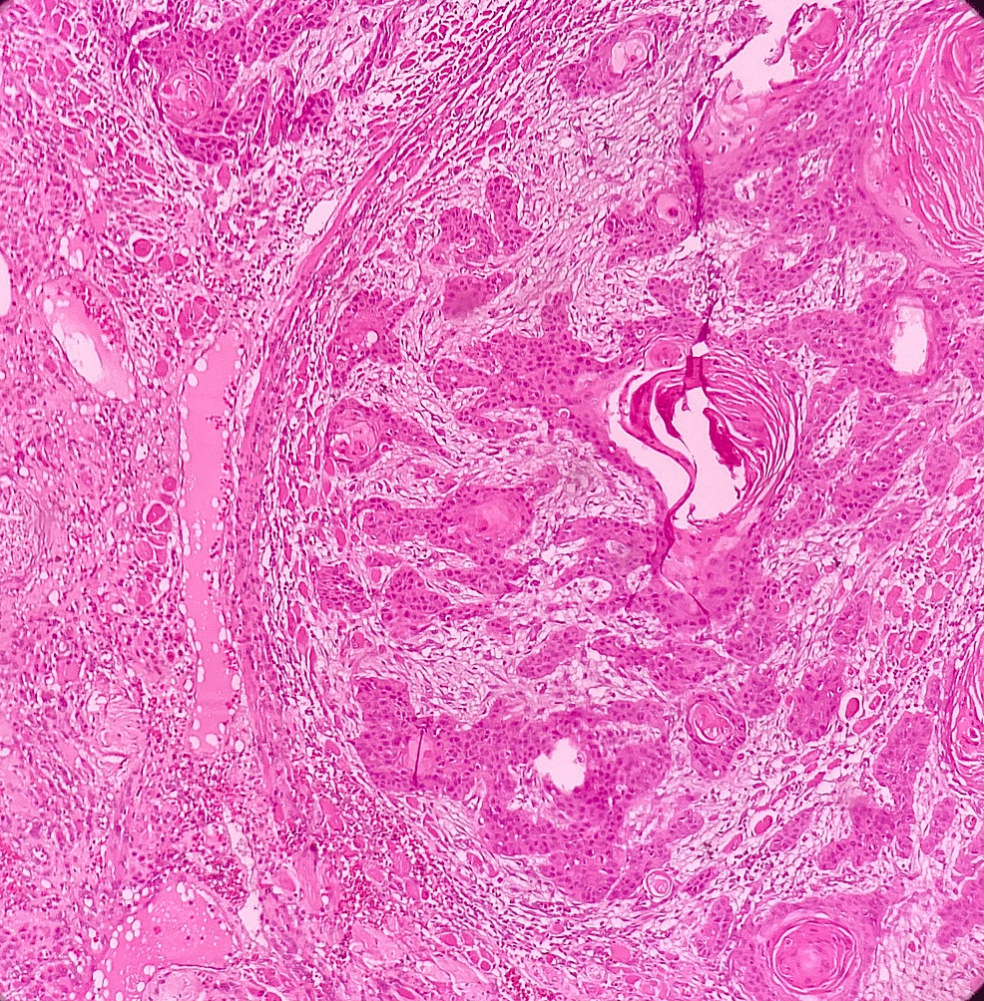
A photomicrograph showing malignant epithelial islands with individual cell keratinization in moderately differentiated squamous cell carcinoma (hematoxylin and eosin, 10x)

**Figure 6:**
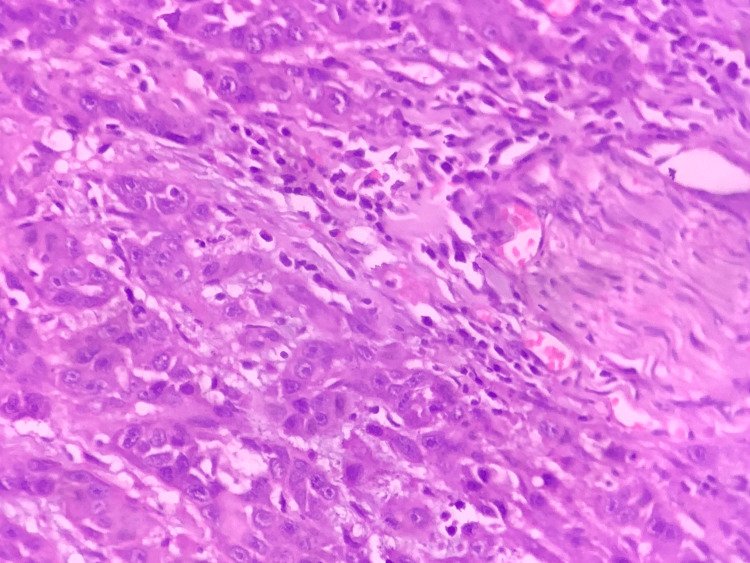
A photomicrograph showing malignant epithelial islands and minimal keratinization in poorly differentiated squamous cell carcinoma (hematoxylin and eosin, 40x)

All of the patients (100%) had squamous cell carcinoma, including one case of spindle cell carcinoma. Forty-nine percent of the cases were well-differentiated squamous cell carcinoma (Figure 4), 23% were moderately differentiated squamous cell carcinoma (Figure 5), and 28% were poorly differentiated squamous cell carcinoma (Figure 6).

**Figure 7:**
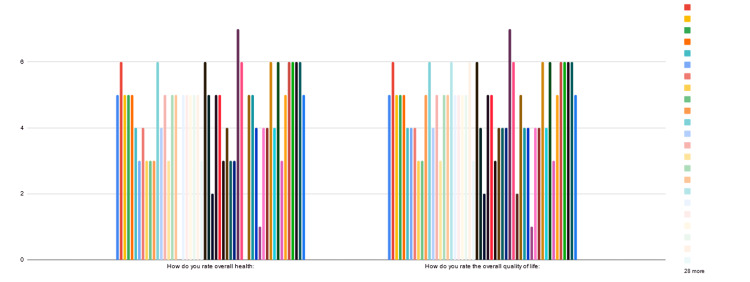
Graphical representation of recorded QOL scores The image has been created by the authors.

Based on QOL findings, our patients had increased pain and difficulties in swallowing, chewing, and speaking. Increased levels of anxiety and concern about appearance and social activities were noted (Figure 7).

**Figure 8:**
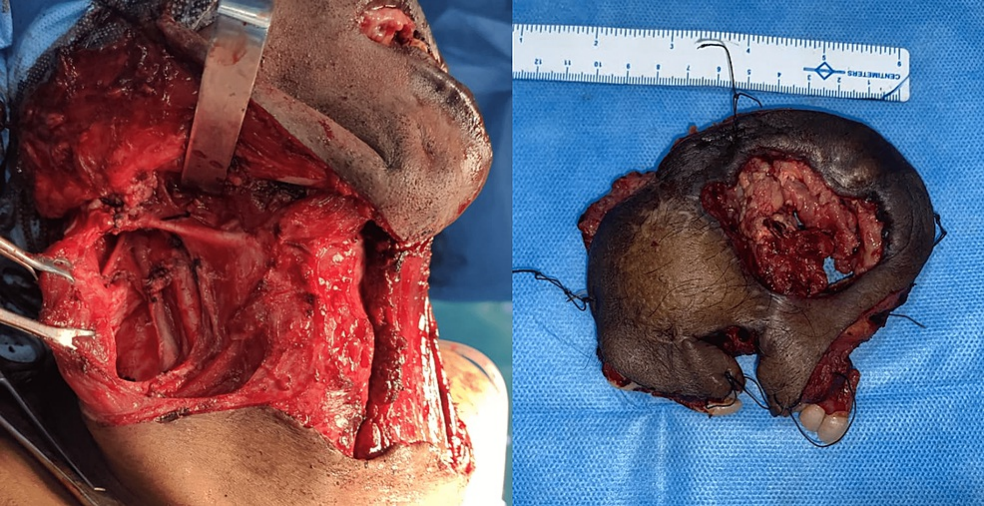
Intraoperative photographs showing the surgical excision with neck dissection (right) and the excised specimen (left)

Surgical management included 82% resection with excision and 18% excision only. The various types of resection done were marginal mandibulectomy, partial glossectomy, subtotal glossectomy, segmental mandibulectomy, rim resection, and partial and total maxillectomy. All the patients underwent neck dissection. Fifty-two percent of the patients underwent supraomohyoid neck dissection, 40% of the patients underwent modified radical neck dissection, and 8% of the patients underwent radial neck dissection. The surgical margins of all the patients were evaluated with cryosections at the time of surgery for tumor clearance (Figure 8).

**Figure 9:**
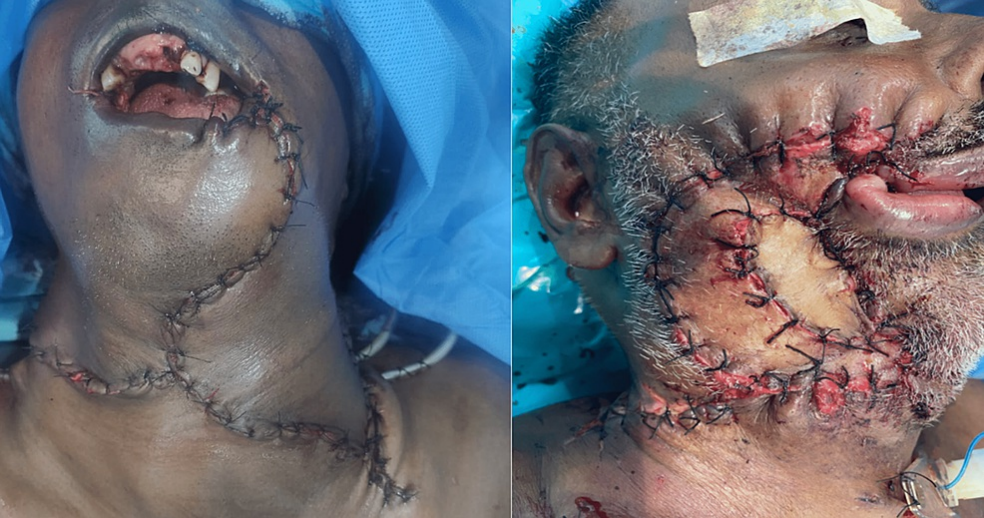
Postoperative pictures of patients after primary closure (right) and reconstruction (left)

Seventy percent of the patients had reconstruction, while the remaining 30% underwent primary closure (Figure 9).

**Figure 10:**
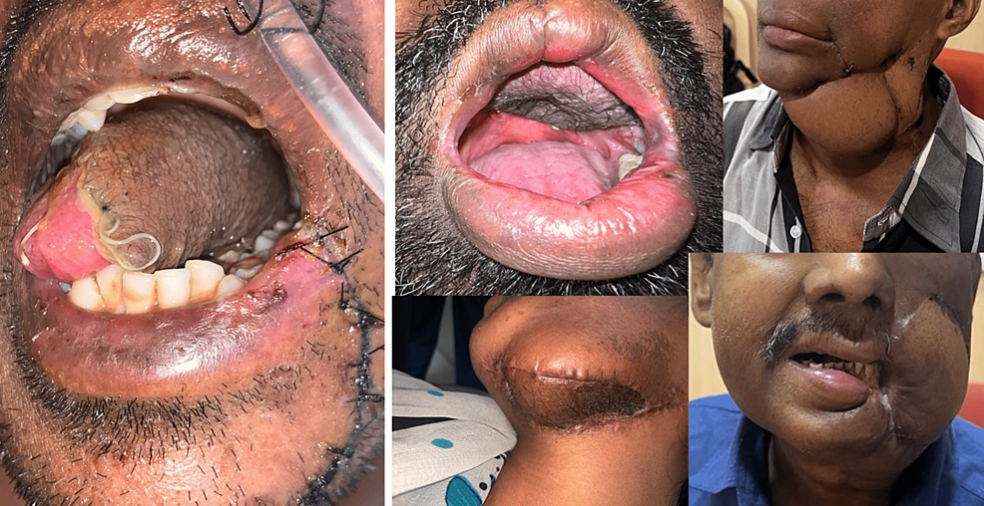
Clinical pictures showing treated patients after one-year follow-up

Postoperative margin clearance was confirmed with excisional biopsy reports for all the cases. Pathological tumor-node metastasis (pTNM) was given to all the patients. All 35 patients were surgically treated and followed up with radiotherapy, chemotherapy, or both. Twenty-six percent of our patients underwent postoperative radiotherapy. Twenty-one percent underwent postoperative chemotherapy, and 15% underwent both protocols. All the patients were followed up for a year after primary treatment (Figure 10).

**Figure 11:**
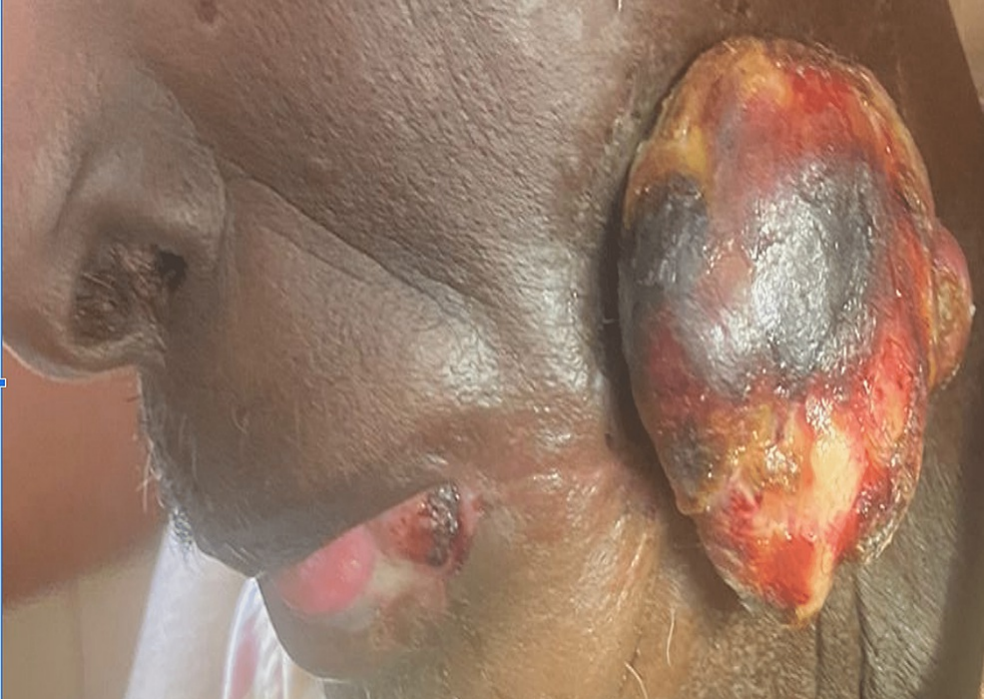
Clinical picture showing a recurrent lesion on the buccal mucosa after primary surgery and postoperative radiotherapy

Out of the 35 included cases, five patients had died (14% mortality). The primary site was buccal mucosa in all five cases. In spite of multiple treatment modalities, three patients had recurrences (Figure 11) post-surgery or post-radiotherapy and succumbed to the disease.

**Figure 12:**
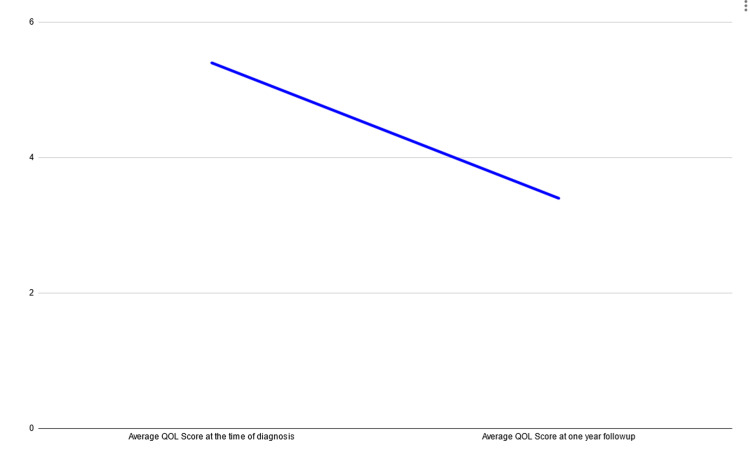
A graph representing the average QOL scores at the time of diagnosis and one year after follow-up The image has been created by the authors.

We observed that the average overall health score and overall QOL score at the time of diagnosis were 5.4. After one year of follow-up, the average rating of overall health and overall QOL was found to be 3.4 (Figure 12).

## Discussion

Oral cancer patients have varied effects post-treatment that impact their QOL. Though there are different treatment modalities available nowadays, patients showed a willingness to compromise. Aoki et al. have reported that the lowest QOL was found immediately after treatment, and it gradually recovered to pre-treatment levels over time [1]. In the scoping review performed by De Cicco et al., only two studies were from India [3].

Hence, our study is one of the pioneers of longitudinal follow-up with QOL estimation in patients treated for oral cancer. This prospective cohort study has revealed that pain, speech, and swallowing were the major domains affected in our patients with oral cancer. There was a consistent drop in the overall health score and quality of life score among the patients who reported having advanced tumors, irrespective of the provided treatment (surgery or radiotherapy) (Table 3).

**Table 3:** Average overall health score and average QOL score recorded at the time of diagnosis and at one-year follow-up

	Time of diagnosis	One-year follow-up
Average overall health score	5.4	3.4
Average QOL score	5.4	3.4

EORTC scoring is given as one to seven, with one (very poor) to seven (excellent) [7]. All of our patients gave the same scores for overall health score and overall QOL score in both instances. Hence, both average scores were the same in our study.

Aoki et al. reported higher anxiety levels before treatment. They also stressed the development of new strategies to compensate for the decline in the head and neck functioning subscale [1]. De Cicco et al. have reported the risk of confounding bias and selection bias in their scoping reviews of various QOL studies. They have also recommended cohort selection based on a composite social class indicator based on three different socio-economic variables: educational level, type of occupation, and household income [3].

The success of oral cancer treatment is not only measured on the basis of the quantity of life but also on the basis of the quality of life, which is an important factor to be measured in OSCC patients. De Cicco et al. have also reported that no scoring system is available that condenses the socio-economic and psychological diagnoses and treatment-specific variables for patients with oral cancer. They have also recommended a longitudinal study design with an evaluation of differences between baseline and post-treatment questionnaires. Baseline QOL could be compromised due to pre-existing comorbidities [3]. Nie et al. stated that their questionnaire design could not assess the quality of eating, and telephonic contacts were not feasible and could not be conducted within a time frame. They also reported that patient responses were not specific to the questionnaire [8].

**Figure 13:**
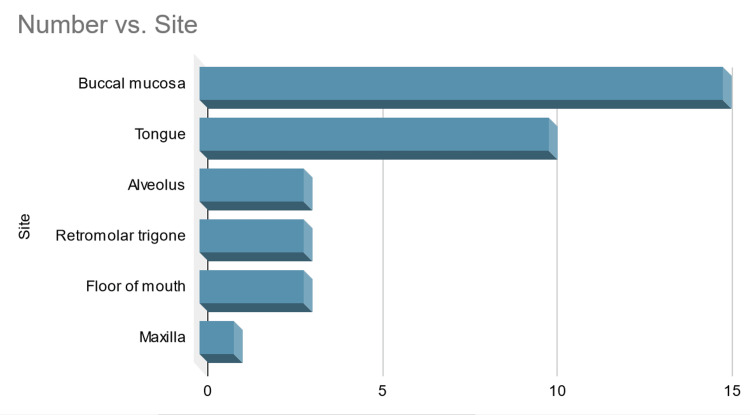
Graphical representation of site involvement observed in our study The image has been created by the authors.

Heutte et al. stated the inherent deficiencies in the generic and specific scales [9]. Meera et al. reported that all the details were not obtained fully due to technical reasons [10]. Nadkarni and Kuderer have independently reported in two large series of cancer patients with COVID-19 that mortality ranged from 13% to 28% [11, 12]. In contrast to the reported literature, we have observed more cases involving the buccal mucosa in our study (Figure 13).

We observed that patients who reported facial skin involvement at the time of diagnosis also showed a rapid decline in QOL. Out of 35 included cases, 15 patients presented with advanced tumors involving buccal mucosa, and five patients died (14% mortality). Three patients also had recurrences post-surgery and post-radiotherapy of the tumors involving buccal mucosa.

It is stated that "it is important to provide preoperative psychological reassurance to increase postoperative adaptation in dealing with psychosocial difficulties, such as feelings of hopelessness and psychological distress resulting from physical handicaps and subsequent to significant life changes" [13].

Surgery in the head and neck region disrupts oral functions, like speech, chewing, and swallowing; sensations, like taste and smell; and social interactions, including communication and expression of interest. Because surgical disfigurement is frequently visible, patients may experience lower self-esteem, lower self-confidence, and body image issues when compared to cancers involving internal organs. Disfigurement is a greater concern for younger patients. Women who underwent disfiguring surgeries had a higher impact on QOL when they did not have adequate social support. Survivors with disfigurement face negative economic and social ramifications, as well as widespread stigma, and research has shown that these effects continue unabated even after a year of surgery [14].

According to various researchers, the growing recognition of QOL as an important outcome of dental care has created a demand for a variety of instruments like the OHIP-14 to measure oral health-related QOL. It is proven to have good reliability, validity, and precision. It is a 14-item test with four main areas of focus: functional restriction, psychosocial disability, pain, and discomfort. The survey and questionnaire used forced selection and Likert scale question forms by Naidu GS et al. [15].

In the cross-sectional study of 34 patients with oral cancer undergoing free flap surgery, Khandelwal et al. reported better QOL in patients with smaller tumors. Patients using feeding tubes had worse results in function [16]. Mair D et al. performed a prospective cohort study on 38 patients with T4 buccal mucosa cancer treated by surgery with ablation, neck dissection, and reconstruction with the Pectoralis Major MyoCutaneous (PMMC) Flap as the first line of treatment. Patients with relapses had a poorer quality of life than those who were disease-free [17].

The QOL is a factor that has to be taken seriously, and personal interaction with every patient has to be done at every review. The QOL significantly deteriorates post-surgery and has to be constantly monitored with adequate motivation and special monitoring of the diet by the clinical nutritionist. Hence, the surgeon should aim for a limited resection followed by the best repair necessary for the patient's age and stage of the disease in order to improve the health-related QOL of the patient. Adequate counselling has to be given; while some patients' primary concern is pain, a few others focus on aesthetics and function.

QOL assessment requires scales that assess generic and specific aspects of these patients. A properly designed, tested, and validated assessment tool that incorporates clinical and histopathological parameters along with patient-reported outcome measures (PROM) and patient-reported experience measures (PREM) would greatly influence the success of the treatment in OSCC patients.

There is no previously published data about the quality of life in our region. Our study is a pilot attempt to use EORTC QLQ-C30 and HN43 in patients with oral cancer. India has a very high incidence of oral cancer. Hence, treatment decisions based on the quality of life, patient-reported outcome measures, and patient-reported experience measures will eventually help in achieving the best outcome.

QOL should be considered a high priority in addition to survival. We have identified that OSCC involving buccal mucosa has low QOL, higher recurrence, and increased mortality in contrast to tongue OSCC widely reported in the literature. Most of our patients had difficulty speaking, difficulty talking with strangers, taste alterations, pain in their jaws and teeth, difficulty swallowing, and problems chewing. Hence, regular follow-up is required, supportive therapy should be initiated whenever the patient has issues with oral function, and continuous monitoring is required to improve their QOL.

Limitations

In our study, we considered only 12 months post-treatment, and further changes in QOL will happen over a longer period. We have also employed EORTC QLQ-HN43 for our study. Other validated QOL scales are also available and have to be tested among the same cohort of patients.

Further studies with larger, more focused samples on various subtypes of OSCC will help validate our findings. A comparison with other validated QOL scales like WHO and OHIP-14 among the same patients will add more valuable information. A longitudinal follow-up study with an assessment of QOL at the time of diagnosis and continuous monitoring post-treatment will provide data to create a treatment model based on QOL.

## Conclusions

The administration of the EORTC QLQ proved to be effective, and this has given us a framework for measuring the quality of life of our oral cancer patients over time. It is a vital tool for a clinician since it enhances patient planning with provisions for proper treatment and supportive care. In our study, we identified a strong correlation between tobacco usage and OSCC.

We are currently pursuing a QOL cohort study on patients with oral cancer who are under continuous follow-up from the time of diagnosis and through various time intervals after treatment (surgery, radiotherapy, and chemotherapy). We are also planning a multi-center cross-sectional study and a multi-center prospective cohort study to identify regional variations in QOL and demographic characteristics of patients with oral cancer.
